# Practical Clinical Training in Skills Labs: Theory and Practice

**DOI:** 10.3205/zma001062

**Published:** 2016-08-15

**Authors:** T. J. Bugaj, C. Nikendei

**Affiliations:** 1 University Hospital Heidelberg, Center for Psychosocial Medicine, Department of General Internal Medicine and Psychosomatics, Heidelberg, Germany

**Keywords:** skills lab, skills lab training, skills, simulation, medical education

## Abstract

Today, skills laboratories or “skills labs”, i.e. specific practical skill training facilities, are a firmly established part of medical education offering the possibility of training clinical procedures in a safe and fault-forging environment prior to real life application at bedside or in the operating room. Skills lab training follows a structured teaching concept, takes place under supervision and in consideration of methodological-didactic concepts, ideally creating an atmosphere that allows the repeated, anxiety- and risk-free practice of targeted skills.

In this selective literature review, the first section is devoted to (I) the development and dissemination of the skills lab concept. There follows (II) an outline of the underlying idea and (III) an analysis of key efficacy factors. Thereafter, (IV) the training method’s effectiveness and transference are illuminated, before (V) the use of student tutors, in the sense of peer-assisted-learning, in skills labs is discussed separately. Finally, (VI) the efficiency of the skills lab concept is analyzed, followed by an outlook on future developments and trends in the field of skills lab training.

## Zusammenfassung

Skills-Labs (dt. „Fertigkeitenlabore“, also spezifische Übungsräume zum Erlernen praktischer Fertigkeiten) sind heute nicht mehr aus der medizinischen Ausbildungslandschaft wegzudenken, denn sie bieten die Möglichkeit, klinische Prozeduren noch vor der realen Anwendung am Krankenbett oder im Operationssaal in sicherer und geschützter Atmosphäre zu erproben. Der Unterricht im Skills-Lab erfolgt strukturiert, supervidiert und unter Berücksichtigung methodisch-didaktischer Rahmenaspekte, sodass im Idealfall das wiederholte, angst- und risikofreie Üben von Fertigkeiten gewährt wird. Die vorliegende selektive Literaturübersicht widmet sich in einem ersten Abschnitt der (I) Entstehung und Verbreitung des Skills-Lab-Konzepts. Es folgt (II) eine Darstellung der zugrundeliegenden Hintergrundidee sowie (III) eine Analyse der wichtigsten Wirksamkeitsfaktoren. Im Anschluss werden zunächst (IV) Effektivität und Transfer dieser Ausbildungsmethode beleuchtet, bevor (V) der Einsatz von studentischen Tutoren im Sinne des peer-assisted-learnings innerhalb des Skills-Labs gesondert darstellt wird. Im Anschluss erfolgt eine Analyse (VI) der Effizienz des Skills-Lab-Konzeptes, bevor in einem letzten Abschnitt ein Ausblick auf zukünftige Entwicklungen und Trends im Bereich der Skills-Labs gegeben wird.

## Introduction

In its broadest sense, the term *“skills labs”*, an abbreviation of skills laboratories, refers to specifically equipped practice rooms functioning as training facilities offering medical students, physicians in training and other medical staff alike a protected, fault-forgiving environment for the practice of clinical skills prior to their real life application. The present selective literature review aims to give the interested reader a brief summary of the scientific knowledge relevant to skills lab training in primary medical education and places the focus specifically on the former target group, namely, on medical students. In light of the fact that the majority of – often serious – mistakes come to effect in the first few working years after medical licensure and can often be put down to a lack of (practical) experience, this group appears to draw particular benefit from skills lab training for several reasons [1]. In addition, after years of mostly theoretically-oriented university education, medical students are only given a relatively short period of time to acquaint themselves with the practical clinical reality in the physician’s professional world. Ultimately, these promising young physicians have a particularly long period of responsibility ahead of them and will in turn be responsible for the training of future health worker generations [1]. Against this background, it seems crucial that clinical procedures are trained in a safe and fault-forgiving environment prior to real life application at bedside or in the operating room. Hence, skills labs play a key role in medical training quality assurance. Here, procedural skills can be trained, repeatedly practiced, and evaluated until the required minimum standard for patient treatment is ensured [2], [3]. Skills lab training provides medical students with the necessary basic skills for later clinical activity by the means of models, phantoms, and fellow students or with the help of standardized patients (SP) [4]. Skills lab training follows a structured teaching concept, takes place under supervision and in consideration of methodological-didactic concepts, ideally creating an atmosphere that allows the repeated, anxiety- and risk-free practice of targeted skills and ensures that all students are given the opportunity to perform these independently.

In this selective literature review, the first section is devoted to

1. the development and dissemination of the skills lab concept. There follows
2. an outline of the underlying idea and (III) an analysis of key efficacy factors. Thereafter,
3. the training method’s effectiveness and transfer are illuminated before
4. the use of student tutors, in the sense of peer-assisted-learning, in skills labs is discussed separately. Finally,
5. the efficiency of the skills lab concept is analyzed, followed by an outlook on future developments and trends in the field of skills lab training.

## Methods

The present paper is based on a “narrative” or “selective” literature review, including author-specific expertise and teaching experience as well as a selective search of thematically relevant references in the database “PubMed”. The key reviews considered are summarized in Table 1 (Tab. 1).

### (I) Development and dissemination

The first skills labs were established at the universities of Illinois and Maastricht in the 1970s with the aim of improving clinical practical skills [5], [6]. Whereon, the use of simulation-based teaching units has seen widespread development in medical education over the last 25 years [7]. Moreover, following the amendments to medical licensure laws in 2002, which allotted key importance to the training and evaluation of clinical skills at medical school, skills labs have been established nationwide and are part of medical education at all medical faculties in Germany [8], [9]. 

#### (II) The underlying idea

Prior to the widespread introduction of skills labs, the acquisition of individual clinical practical skills, and hence successful medical education, was strongly dependent on “appropriate” patient encounters and well-trained lecturers [10]. Since access to these may be severely limited by availability and time problems [11], skills lab training provides a comparatively simple and almost always-available solution to the sketched dilemma. Furthermore, technical innovations in diagnosis and treatment, as well as large-scale patient safety initiatives have changed the demands on physicians’ skills over the past years, giving even further justification to skills lab training [12], [7]. For instance, technically sophisticated innovations, such as minimally invasive surgery, require the specialized training of psychomotor and spatial awareness skills. In a study from 2003, Ziv et al. point out that the use of simulation-based training in medical education, similar to its use in the fields of aviation or the military, represents, as it were, an ethical imperative and therefore see the acquisition of a basic routine prior to “real patient practice” as an indispensable step in medical training [13]. Hence, simulation-based medicine not only allows for the training of rare emergency measures but is, in itself, a crucial prerequisite to ensure good practice. Especially, the aspect of patient safety is of particular importance here: situations handled by medical students or young physicians with lacking adequate prior medical training have been shown to increase patient risk [14], [15]. Accordingly, in a study from 2006, Takayasu and colleagues were able to show that students particularly appreciated the aspect of experiential “practice without risk” of harming real patients in simulation-based training [16]. In addition to the provision of a fault-forgiving environment, professional feedback in particular is seen to make skills lab training a valuable experience for students. Accordingly, this important aspect and its influence on behavior are discussed separately below.

#### (III) Key efficacy factors

A Best Evidence Medical Education (BEME) collaboration review from 2005 [17] offers a list of key efficacy factors for simulation-based training in medical education, which also come to effect in skills lab training. For example, the importance of giving feedback directly during actual simulation-based training is emphasized. Furthermore, in line with the principle of “deliberate practice”, it is mentioned that repeated simulation-based practice is necessary in order to achieve best results. The reflections upon the required degree of realism, as in how close to real-life practice should and simulation training can come, also seem relevant in the establishment of skills lab training units. In addition to requiring coherent integration into the existing curriculum, simulation-based learning should take place in a “controlled” environment. Moreover, in accordance with the BEME guidelines, it is important that simulated skill performance outcome and assessment are measured in a standardized and clearly defined way. All mentioned points shall be discussed below in detail and the said BEME collaboration review key efficacy factors will be enhanced by some further important factors. Accordingly, a section on skills lab training preparation as well as a passage on standardization going beyond the simple definition of learning objectives by, for example, also considering the importance of unified teaching concepts and discussing appropriate assessment methods, can be found below. Finally, it should not go unmentioned that precisely the implementation of these key efficacy factors can often be held responsible for high costs, financial burden or problems related to skills labs operation or establishment.

##### Setting

The skills lab setting allows students to make mistakes and ideally to discover and correct these accordingly without having to fear adverse consequences for themselves or patients [17]. Accordingly, trainers also need not worry about real patients’ well-being during training and can thus concentrate on the skill development of individual trainees, for example, by highlighting “Teachable Moments” [15]. In addition to this “ideal environment” largely shaped by and attributable to the trainers attitude, there are also structural measures that make a skills lab a prosperous practice field and thus contribute to the establishment of an ideal learning setting in a wider sense: appropriately, todays skills labs often reflect the real-life medical working environment. This is achieved by, for example, furnishing the skills labs with identical or comparable equipment and facilities. Some skills labs also have adjoining observation rooms or installed cameras that allow the trainers to follow what is happening inside via a one-way mirror or monitor.

##### Standardization 

In order to learn a practical skill, it is important that learners go through a standardized, trainer-led training program. In this initial phase, free practice time does not seem to be conducive [18]. A coherent didactic concept, such as standard instruction in accordance with Peyton (see below), can be seen as a valid standardized approach in this context. Furthermore, checklists have been shown to be conducive for the quality assurance of students' skills lab education [19] also including internal faculty standards that define examination procedures and principles [20], [21], [22]. Ultimately, the process of standardization provides the basis upon which competence-based assessment of student skills is made possible.

##### Definition of learning objectives

For the acquisition of procedural skills, learners generally have a larger chance of learning success if the learning objectives are clearly defined and the desired outcome has been clearly communicated in advance [17]. As a result, students are given the possibility to prepare themselves meticulously prior to skills lab training. However, the clear definition of learning objectives is also of essential interest to curriculum planning, as they can serve as blueprints for the appropriate definition of the necessary time frame under the careful consideration of the specific requirements and complexity of the particular activity and the learners’ previous experiences. The consensus statement “Practical Skills in Medical Studies” distinguishes three depth dimensions of learning objectives, which seem to be relevant for skills lab learning [23]:

1. To have seen the implementation of the skill
2. To have completed a skill itself several times under supervision
3. To be able to perform a skill independently and routinely

##### Training preparation

In order to maximize the learning effect and to make optimal use of the often limited training periods, skills lab lessons must be specifically prepared in addition to their above-mentioned general integration into the curriculum. To this effect, students are frequently provided with preparatory texts in paper or electronic form in advance. However, other forms of student preparation have also been reported, such as the targeted analysis of virtual patients by the means of multimedia case studies [24].

##### Instruction

During skills lab training, learners commonly practice the procedural skills’ psychomotor component under the trainers’ instruction, who have previously demonstrated the relevant skill. Subsequently, the skills are then performed by the learners themselves under supervision. Peyton’s Four-Step Approach has proven to be most helpful here. This systematic approach to learning practical skills has been increasingly used in the field of medical education and is also the routine approach in the European Society of Cardiology’s (ESC) practical training courses since 2000 [25], [26], [27], [28]. The Four-Step Approach consists of the following four clearly defined steps:

1. The trainer demonstrates the skill in real time without giving instructions or explanatory words (“Demonstration”).
2. The trainer repeats the procedure, this time describing all necessary sub-steps (“Deconstruction”).
3. The trainer performs the skill for a third time, this time following the sub-steps only as described to him by the trainee (“Comprehension”). This step has been identified as the most important step of the Four-Step Approach in the past [29] as deeper processing mechanisms reflecting what was observed in the first two steps are necessary for the trainees’ to be able to give instructions.
4. The trainee performs the skill on his/her own (“Performance”).

Compared to the provision of simple instructions, Peyton’s approach has been shown to be more conducive, especially, in regard to observed professionalism and concomitant doctor-patient communication and also leads to the more rapid implementation of new activities [29], [30]. Though the procedure described above focuses a one-on-one learning situation, a modified version of the approach has also proven to be applicable for the instruction of small groups, typically found in skills lab training settings [31].

##### Deliberate Practice

First described by K. Anders Ericsson in 1993, the idea of “Deliberate Practice” (DP) was initially derived from various studies with professional musicians, as the relationship between invested practice and achieved level of musical excellence presented itself particularly for empirically investigation [32]. Previously, it was often assumed that experts have innate skills not possessed by other people. However, according to Ericsson these skills are not innate or unchangeable but the result of lifelong and especially deliberate, as in systematic and goal-oriented, practice of an activity [32]. DP involves (a) repetitive practice of the intended skill, combined with (b) the thorough assessment of the skill so that the learner (c) can receive specific, informative feedback, which results in an increasingly (d) better performance of skill. So, according to Ericsson et al., the improved performance of an activity largely depends on how much time one spends actively practicing it – time alone does not suffice to achieve expert status. Even for skills of little complexity, repetitive practice seems very important [33] and is even indispensable for medium or highly-complex skills [34]. Hence, complex skills, for example in the field of anesthesia (plexus block, spinal or epidural anesthesia), have been demonstrated to require a significantly higher number of repetitions to achieve mastery [35]. DP therefore appears to play a major role in the learning of skills and thus is a key impact factor in skills lab training. However, it has been argued that fundamental basic skills could well be hereditary and that other factors, such as general cognitive abilities, are also conducive in achieving high skill performance [36], [37]. In order to ascertain the extent to which individual students learn using DP methods, Moulaert et al. conducted a survey with medical students. Subsequently, the authors compared the questionnaire study’s results with the students’ university performance. They were not only able to discover a relationship between DP and the academic performance but were also able to show that the high-performing students were more likely to practice DP learning than the lower performers [38]. In 2011, Duvivier et al. showed that students’ OSCE results improved with the increasing use of DP methods [39]. In turn, Griswold and colleagues were able to confirm that the DP method also improved practical skills increasing patient safety consecutively [14]. Moreover, McGhie even reported a strong dose-response relationship: the number of hours subjects spent practicing with high-fidelity simulators correlated with standardized learning outcomes [40].

##### Feedback

Issenberg et al. list feedback, that is the knowledge of the personal performance in the carried out simulation, as the most important factor for effective simulation-based learning [17], which can either be generated by the simulator itself (for example “high-fidelity” simulators, as used in the fields of anesthesia and emergency medicine sporting a high degree of realism, are able to simulate a variety of vital signs) or be given by the trainer or other trainees in the vicinity in “real time”. In addition, post hoc feedback by viewing a recorded video footage of the performed skill also represents a possible feedback method [17]. A distinction is generally made between formative and summative feedback. In the context of simulation-based medical training, formative feedback is of higher priority, as it is usually not a question of “pass” or “fail” but of the trainees’ skills improvement [41]. Feedback helps trainees to reflect upon their own actions, to recognize possible errors and to subjectively evaluate and observe their learning progress [17]. Just recently, Bosse et al. were able to show that higher-frequency intermittent feedback is superior to low-frequency feedback for the acquisition of procedural skills during skills training [42]. However, other research has indicated that too intensive feedback can be inconducive, especially in the early stages of learning processes [43].

##### Fidelity

In regard to the effectiveness of skills lab training, the importance of the degree of the simulations’ realism has been controversially discussed: while some studies have found evidence that the degree of realism achieved in a simulation positively impacts on learning effect [17], [44], others argue that the learners‘ motivation to engage in the simulation and to complement the fictitious situation by augmenting it with personal real-life experiences are further key factors in addition to the simulation’s fidelity [45]. Accordingly, other studies have shown no or only minimal training outcome advantages for high-fidelity simulation settings, hardly justifying the ensued considerable extra costs [46], [47], [48]. However, in order to integrate the implementation of procedural skills into a well-functioning role play, the provision of role assignments, role-specific instructions and case vignettes is crucial in skills lab training [19], [49]. A controlled study was able to show that the introduction of role play into skills lab training resulted in both a more realistic perception of the medical role, as well as in more intensive and improved communication accompanying the procedures [19]. Today, Miller’s Learning Pyramid is an often-cited guide to describe different levels of knowledge and skills acquisition [50] which distinguishes four levels of competence or training objectives:

1. Knows
2. Knows how
3. Shows how
4. Does

Accordingly, the first two levels describe cognitive aspects, i.e. the acquisition of factual knowledge (level 1) and the application knowledge (level 2). The third and fourth levels refer to procedural skills. Skills lab training takes place on Miller’s Pyramid’s 3^rd^ competence level, in detail on the level “shows how”/“show as if”. The acquisition of procedural skills in turn can take place in simulated environments with a varying degree of realism. In a publication from 2013, Russo and Nickel stress (49) that there is no such thing as an ideal degree of realism for simulations in medical training but that, depending on the training’s focus and simulators, suitable conditions for the rehearsal of specific action or movement sequences can be achieved with a relatively low degree of fidelity. However, the authors of this paper believe that even a good simulator, be it in combination with other factors aiming to further increase its degree of realism, such as accompanying role play, fails to achieve Miller’s fourth level of competence (“Does”) as no simulation can fully reflect and thus endeavor to replace real-life practical action. 

##### Curriculum integration

In 2011, Parmar et al. reported that ideal skills lab training is most likely to be dependent on a combination of the provision of optimum curriculum in conjunction with a suitable phantom [51]. The above-mentioned BEME Collaboration overview from 2005 [17] also implies that simulation-based learning should not be seen as an optional offer but as an integral (and standardized) part of medical education, particularly as optional services, such as elective subjects, usually arouse much less interest among students [17]. Furthermore, in a study from 2012, Weller and colleagues come to the conclusion that simulated practice should necessarily be in temporal association with practical teaching at bedside allowing for the direct transference and consolidation of learned skills in practice [15]. The more complex the simulation, the greater the risk of frustrating or discouraging students, especially when practical skills lab training is not embedded in a coherent overall curriculum or adapted to the level of training [52]. Hence, simulation-based training should be deemed to be of equivalent importance as bedside teaching, lectures and/or problem-oriented learning, which is a challenge for curriculum development [41]. Already in 2009, Kneebone had indicated that the acquisition of practical skills should not be completely separated from the clinical context and that an excessive simplification of highly complex activities could lead to a superficial understanding in learners [53]. Therefore, it seems all the more important to find the right balance between simulation-based training and clinical reality in a commensurate curriculum conducive to learning.

##### Linking the skills lab training with practical clinical examinations

Depending on the focus, examinations represent a specific learning incentive to acquire theoretical and practical knowledge or manually-practical skills. The phenomenon known as “assessment drives learning” [54], [55] can be harnessed by creating congruence between the learning and examination format within the respective competence levels (knows, knows how; shows how; does; cf. Miller’s Pyramid [50]). Such “constructive alignment” [56] is achieved, for example, if the set-up of a peripheral IV catheter is practiced in skills lab training (level “shows how”) and the degree of competence acquired is then evaluated via a practical, not theoretical, examination on the same level of competency [57]: hence, the examination format adequately corresponds with the learning level. It has been shown that skills lab training learning content relevant for OSCE examinations (objective structured clinical examination) was continuously and increasingly practiced during the semester itself, whereas training opportunities without relevance to assessment were no longer made use of towards the end the semester [58].

#### (IV) Effectiveness and transfer in skills labs training

Given the increase in simulation-based training opportunities in medical education, the question as to the effectiveness and transference of practiced skills in skills lab training arises. In 1999, Remmen et al. [59] showed that skills labs training increased the number of independently performed skills by medical students in everyday clinical practice, although the validity of the results is weakened by the small participant number. Also, the quality of the underlying skills was not considered. In 2005 in a prospective study, Jünger et al. [60] studied the impact of basic practical internal medicine skill training (incl. seven units of skills lab training lasting 90 min. each) on students‘ OSCE examination performance. The intervention group performed significantly better in the OSCE examination than the control group, which had only received classic bedside training. In 2012, Lund et al. were able to show that skills lab training not only led to an improved examination performance in practical clinical trials but also to an objective and significantly higher rated transfer capacity for clinical skills acquired in skills lab training (equivalent to the highest level of Kirkpatrick’s hierarchy for the evaluation of training effects [61]) via the example of the set-up of a plastic cannula [62]. 

In addition, several reviews and meta-analyzes on the effectiveness of skills lab training exist: in a review from 2007, Lynagh et al. [63], examined transference to the clinical reality in addition to the question of the effectiveness of skills lab training. Unfortunately, only 20 of the 44 studies included in the review considered the transfer of procedural skills. In turn, eight of the 20 studies used animal carcasses or live animals instead of real patients. Therefore, these studies are, strictly speaking, not able to answer the question of transfer to reality but rather address the question of transference of low fidelity simulations to high-fidelity simulations. However, of the twelve studies that actually examined the transference of procedural skills to clinical reality, eleven studies found that participants who had received a simulation-based training intervention significantly improved in performance compared to the control group. The research group led by Cook and colleagues contributed several important meta-analysis to assess the effectiveness simulation-based training in medicine: in 2011, Cook et al. [64] were able to show that simulation-based interventions were superior to no intervention settings, stressing that no further primary studies of this kind would be necessary as the large effect sizes in the areas of knowledge, attitude and skills and moderate effects on patient care outcome had been clearly proven for simulation-based training. In 2012, in a further meta-analysis, Cook and colleagues examined the superiority of simulation-based training in medicine over other instruction methods and could substantiate small to moderate effects over training forms which had dispensed simulation use [65]. In a meta-analysis in 2011, McGhie et al. [66] also investigated the effect of simulation-based training in medicine using Deliberate Practice (see above) compared to traditional teaching methods and were able to demonstrate its superiority, although they, similar to Cook et al., noted the relative lack of high-quality studies on the effectiveness of simulation-based teaching in medicine. In a randomized controlled trial in 2013, Hermann-Werner and colleagues were able to show that skills lab training learning effects also lasted longer than it seems to be the case with traditional teaching methods [67]. In this study, participants who received skills lab training performed the observed activities (insertion of a peripheral IV catheter and a gastric tube on a model) significantly better as compared to students who had previously only received training in a “see one – do one” intervention. However, this observation was not only limited to immediately after exercise; in follow-ups three or six months later, skills lab intervention group students required less time to carry out the skills and were also significantly more likely to be rated as clinically competent as compared to students that had been traditionally instructed.

#### (V) Skills lab training and peer-assisted learning

Skills lab training must not necessarily be led by medical faculty in order to yield long-term success. Moreover, implementing training by specially trained student tutors is quite widespread: according to a recent study published by Blohm et al. the principle of the PAL (peer-assisted learning) is used in about 90% of the skills labs of the surveyed German faculties. This could be down to the fact that several studies have been able to show that PAL is equivalent to medical faculty instructed training [68], [69], [70] as it allows for “eye level learning” [58], [71]. Depending on the focus, students’ cognitive, psychomotor and affective development has been proven to be supported by PAL [72]. Social and cognitive congruence between tutors and students have often been listed as key PAL efficiency factors [73], [74]. The construct of social congruence means that student tutors and students are able to communicate informally and in a particularly empathic manner simply because of their similar social roles [75]. In addition, student tutors and students often share a similar knowledge base and comparable learning experiences, commonly termed “cognitive congruence”. Accordingly, both speak the same “language” facilitating the delivery of explanations at an appropriate level [71].

#### (VI) Skills-Labs training efficiency 

Regardless of the above-discussed question of effectiveness, the question of this measure’s efficiency must inevitably arise. The obvious question of whether the – in some cases very high – effort and resource investment required to achieve the objective is justifiable is at the heart of this deliberation. Accordingly, the methods high cost is also the most frequently mentioned criticism of the concept of simulation-based training in medicine [76]. The exact cost of skills lab training is not easy to elicit, as costs reports are often irregular and / or incomplete according to Zendejas et al. observations [77]. The same meta-analysis from 2013 shows that from a total of 967 identified comparative studies only a fraction (6.1%) even mention the cost of the underlying intervention, with a median of two reported cost factors, although the meta-analysis' authors identify 21 relevant factors. These figures clearly underline significant deficiencies in the report of simulation costs. Only 1.6% of all studies identified by Zendejas et al. compared the cost of the simulation to those of different training forms. The authors of the meta-analysis list expenditure on simulators and accessories as the most commonly cited cost factors, followed by spending on consumables and simulation unit maintenance fees. However, the costs for room rental, furniture and employed tutors' wages are often overseen. Regardless of the exact cost, it is a already well-established fact that medical education is a particularly expensive field of education [78], which may be largely attributable to practical training. Hence, skills labs training should never be implemented as a mere end in itself but should always be subjected to the strict scrutiny of whether the intended learning objective can be reached by the means of other teaching methods, or if the use of simulators offers additional benefit, such as ensuring patient safety. Accordingly, deliberations on cost efficiency must consider that the renunciation of simulation-based training and the return to exclusive training "at bedside" may potentially ensue much higher costs due to the potential harm to patients [79]. Regardless of the obvious and aforementioned ethical concerns, several authors have also noted that the use of real patients for training is by no means inexpensive [80], [81], [82]: unlike in simulation-based training, materials, such as a catheter, rendered unsterile by careless actions can not be reused, therefore, increased material costs must be anticipated. Moreover, without prior simulator-based training, students require more time to carry out skills, which can cause problems even ensuing more costs in the usually tight schedules of clinics. Viewed differently, by offloading time-consuming teaching content from the clinical routine, simulations can also be seen to provide a possibility of saving cost not to be underestimated. The application of a skill in a real-life setting by a learner often ensues a higher level of iatrogenic injuries, which, in turn, require costly treatment.

## Outlook

In 2002, the amendment of the medical licensing regulations already launched a number of developments in national medical education towards the more focused practical training of future physicians [https://www.gesetze-im-internet.de/_appro_2002/BJNR240500002.html]. Today, a further shift in curriculum content in favor of communicative and practical clinical skills training is highly probable in light of the introduction of the “National Competency-based Catalogue of Learning Objectives for Medical Education” [83] and the future “National Longitudinal Core Curriculum of Communications in Medicine”. In addition, the “Master Plan 2020” lists further measures promoting practical relevance in medical education [https://www.bundesgesundheitsministerium.de/ministerium/meldungen/2015/masterplan-medizinstudium-2020.html]. Consequently, medical education will continue to avert from the outdated model of “see-one, do-one, teach one” and, in all probability, skills lab training will increasingly advance to be a worldwide norm. In addition to further developments in simulation-based learning and training, an increase in methodologically convincing, randomized-controlled, double-blind studies investigating the effectiveness and transference of the procedural skills learned in skills lab in clinical practice as well as the associated costs, can be expected. Nevertheless, these conducive developments in the field of medical education, should never let us forget that simulation-based training always remains an approximation of real-life practice and can never replace real-life patient practice. Hence, even the most modern skills labs and the expected innovations in simulation-based medical education must always be seen as an essential complement – but can never be understood as a complete replacement of quality teaching at bedside and of supervision by experienced lecturers. Notwithstanding the foregoing, medical education today is no longer conceivable without the numerous opportunities and benefits of simulation-based training in skills labs only hinted at in this present work.

## Competing interests

The authors declare that they have no competing interests.

## Figures and Tables

**Table 1:**
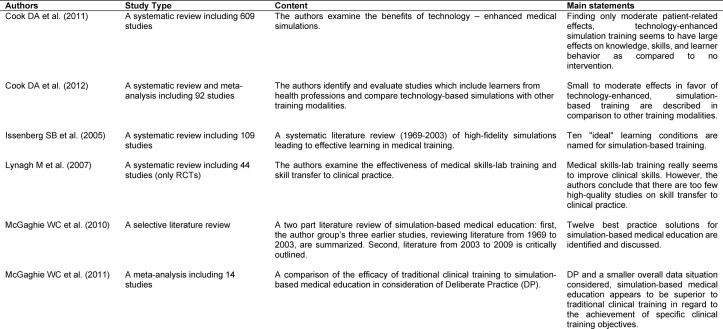
Important skills-lab training reviews in medical education.

## References

[1] Datta R, Upadhyay K, Jaideep C (2012). Simulation and its role in medical education. Med J Armed Forces India.

[2] Du Boulay C, Medway C (1999). The clinical skills resource: a review of current practice. Med Educ.

[3] Bradley P, Postlethwaite K (2003). Setting up a clinical skills learning facility. Med Educ.

[4] Barrows HS (1993). An overview of the uses of standardized patients for teaching and evaluating clinical skills. AAMC. Acad Med.

[5] Sajid A, Lipson LF, Telder V (1975). A simulation laboratory for medical education. J Med Educ.

[6] Van Dalen J, Bartholomeus P, Bender W, Hiemstra R, Scherpbier A, Zwiestra R (1990). Training clinical competence in a skills laboratory. Teaching and Assessing Clinical Competence.

[7] Issenberg SB, Scalese RJ (2008). Simulation in health care education. Perspect Bio Med.

[8] Kruppa E, Junger J, Nikendei C (2009). Innovative teaching and examination methods--taking stock at German medical faculties. Dtsch Med Wochenschr.

[9] Blohm M, Lauter J, Branchereau S, Krautter M, Kohl-Hackert N, Jünger J, Herzog W, Nikendei C (2015). "Peer-assisted learning" (PAL) in the Skills-Lab--an inventory at the medical faculties of the Federal Republic of Germany. GMS Z Med Ausbild.

[10] Scalese RJ, Obeso VT, Issenberg SB (2008). Simulation technology for skills training and competency assessment in medical education. J Gen Int Med.

[11] Gordon JA, Pawlowski J (2002). Education on-demand: the development of a simulator-based medical education service. Acad Med.

[12] Maran NJ, Glavin RJ (2003). Low- to high-fidelity simulation - a continuum of medical education?. Med Educ.

[13] Ziv A, Wolpe PR, Small SD, Glick S (2003). Simulation-based medical education: an ethical imperative. Acad Med.

[14] Griswold S, Ponnuru S, Nishisaki A, Szyld D, Davenport M, Deutsch ES, Nadkarni V (2012). The emerging role of simulation education to achieve patient safety: translating deliberate practice and debriefing to save lives. Pediatr Clin North Am.

[15] Weller JM, Nestel D, Marshall SD, Brooks PM, Conn JJ (2012). Simulation in clinical teaching and learning. Med J Aust.

[16] Takayesu JK, Farrell SE, Evans AJ, Sullivan JE, Pawlowski JB, Gordon JA (2006). How do clinical clerkship students experience simulator-based teaching? A qualitative analysis. Sim Healthcare.

[17] Issenberg SB, McGaghie WC, Petrusa ER, Lee Gordon D, Scalese RJ (2005). Features and uses of high-fidelity medical simulations that lead to effective learning: a BEME systematic review. Med Teach.

[18] Fichtner A, Pierre M, Breuer G (2013). Lernen für die Praxis: Das Skills-Lab. Simulation in der Medizin.

[19] Nikendei C, Zeuch A, Dieckmann P, Roth C, Schafer S, Volkl M, Schellberg D, Herzog W, Jünger J (2005). Role-playing for more realistic technical skills training. Med Teach.

[20] Pjontek R, Scheibe F, Tabatabai J (2013). Heidelberger Standarduntersuchung.

[21] Nikendei C, Kadmon M (2015). Heidelberger Standardprozeduren.

[22] Nikendei C, Ganschow P, Groener JB, Huwendiek S, Köchel A, Köhl-Hackert N, Pjontek R, Rodrian J, Scheibe F, Stadler AK, Steiner T, Stiepak J, Tabatabai J, Utz A, Kadmon M (2016). "Heidelberg standard examination" and "Heidelberg standard procedures" - Development of faculty-wide standards for physical examination techniques and clinical procedures in undergraduate medical education.. GMS J Med Educ.

[23] Schnabel KP, Boldt PD, Breuer G, Fichtner A, Karsten G, Kujumdshiev S, Schmidts M, Stosch C (2011). A consensus statement on practical skills in medical school - a position paper by the GMA Committee on Practical Skills. GMS Z Med Ausbild.

[24] Lehmann R, Bosse HM, Huwendiek S (2010). Blended learning using virtual patients and skills laboratory training. Med Educ.

[25] Bullock I (2000). Skill acquisition in resuscitation. Resuscitation.

[26] Bullock I, Davis M, Lockey A, Mackway-Jones K (2015). Pocket Guide to Teaching for Clinical Instructors.

[27] American Heart Association (2001). R. C. Instructor's manual: advanced cardiovascular life support.

[28] Nolan J, Baskett P, Gabbott D (2001). Advanced life support course provider manual.

[29] Krautter M, Weyrich P, Schultz JH, Buss SJ, Maatouk I, Junger J, Nikendei C (2011). Effects of Peyton's four-step approach on objective performance measures in technical skills training: a controlled trial. Teach Learn Med.

[30] Krautter M, Dittrich R, Safi A, Krautter J, Maatouk I, Moeltner A, Nikendei C (2015). Peyton's four-step approach: differential effects of single instructional steps on procedural and memory performance - a clarification study. Adv Med Educ Pract.

[31] Nikendei C, Huber J, Stiepak J, Huhn D, Lauter J, Herzog W, Jünger J, Krautter M (2014). Modification of Peyton's four-step approach for small group teaching - a descriptive study. BMC Med Educ.

[32] Ericsson KA, Krampe RT, Tesch-Römer C (1993). The role of deliberate practice in the acquisition of expert performance. Psychol Rev.

[33] Lammers RL (2008). Learning and retention rates after training in posterior epistaxis management. Acad Med.

[34] Ericsson KA (2007). An expert-performance perspective of research on medical expertise: the study of clinical performance. Med Educ.

[35] Konrad C, Schupfer G, Wietlisbach M, Gerber H (1998). Learning manual skills in anesthesiology: Is there a recommended number of cases for anesthetic procedures?. Anesth Analgesia.

[36] Hambrick DZ, Meinz EJ (2011). Limits on the predictive power of domain-specific experience and knowledge in skilled performance. Curr Direct Psychol Sci.

[37] Campitelli G, Gobet F (2011). Deliberate Practice Necessary But Not Sufficient. Curr Direct Psychol Sci.

[38] Moulaert V, Verwijnen MG, Rikers R, Scherpbier AJ (2004). The effects of deliberate practice in undergraduate medical education. Med Educ.

[39] Duvivier RJ, van Dalen J, Muijtjens AM, Moulaert VR, van der Vleuten CP, Scherpbier AJ (2011). The role of deliberate practice in the acquisition of clinical skills. BMC Med Educ.

[40] McGaghie WC, Issenberg SB, Petrusa ER, Scalese RJ (2006). Effect of practice on standardised learning outcomes in simulation-based medical education. Med Educ.

[41] McGaghie WC, Issenberg SB, Petrusa ER, Scalese RJ (2010). A critical review of simulation-based medical education research: 2003-2009. Med Educ.

[42] Bosse HM, Mohr J, Buss B, Krautter M, Weyrich P, Herzog W, Jünger J, Nikendei C (2015). The benefit of repetitive skills training and frequency of expert feedback in the early acquisition of procedural skills. BMC Med Educ.

[43] Stefanidis D, Korndorffer JR, Jr, Heniford BT, Scott DJ (2007). Limited feedback and video tutorials optimize learning and resource utilization during laparoscopic simulator training. Surgery.

[44] Brydges R, Carnahan H, Rose D, Rose L, Dubrowski A (2010). Coordinating progressive levels of simulation fidelity to maximize educational benefit. Acad Med.

[45] Dieckmann P (2005). "Ein bisschen wirkliche Echtheit simulieren": über Simulatorsettings in der Anästhesiologie.

[46] De Giovanni D, Roberts T, Norman G (2009). Relative effectiveness of high- versus low-fidelity simulation in learning heart sounds. Med Educ.

[47] Grober ED, Hamstra SJ, Wanzel KR, Reznick RK, Matsumoto ED, Sidhu RS, Jarvi KA (2004). The educational impact of bench model fidelity on the acquisition of technical skill: the use of clinically relevant outcome measures. Ann Surg.

[48] Matsumoto ED, Hamstra SJ, Radomski SB, Cusimano MD (2002). The effect of bench model fidelity on endourological skills: a randomized controlled study. J Urology.

[49] Dieckmann P, Rall M, Eich C, Schnabel K, Junger J, Nikendei C (2008). Role playing as an essential element of simulation procedures in medicine. Z Evid Fortbild Qual Gesundheitswes.

[50] Miller GE (1990). The assessment of clinical skills/competence/performance. Acad Med.

[51] Parmar S, Delaney CP (2011). The role of proximate feedback in skills training. Surgeon.

[52] Russo SG, Nickel EA, Pierre M, Breuer G (2013). Wie im wahren Leben: Simulation und Realitätsnähe. Simulation in der Medizin.

[53] Kneebone R (2009). Perspective: Simulation and transformational change: the paradox of expertise. Acad Med.

[54] Muijtjens AM, Hoogenboom RJ, Verwijnen GM, Van Der Vleuten CP (1998). Relative or Absolute Standards in Assessing Medical Knowledge Using Progress Tests. Adv Health Sci Educ.

[55] McLachlan JC (2006). The relationship between assessment and learning. Med Educ.

[56] Biggs J (1999). What the student does: teaching for enhanced learning. High Educ Res Develop.

[57] Wass V, Van der Vleuten C, Shatzer J, Jones R (2001). Assessment of clinical competence. Lancet.

[58] Buss B, Krautter M, Moltner A, Weyrich P, Werner A, Junger J, Nikendei C (2012). Can the 'assessment drives learning' effect be detected in clinical skills training?--implications for curriculum design and resource planning. GMS Z Med Ausbild.

[59] Remmen R, Derese A, Scherpbier A, Denekens J, Hermann I, van der Vleuten C, Van Royen P, Bossaert L (1999). Can medical schools rely on clerkships to train students in basic clinical skills?. Med Educ.

[60] Junger J, Schafer S, Roth C, Schellberg D, Friedman Ben-David M, Nikendei C (2005). Effects of basic clinical skills training on objective structured clinical examination performance. Med Educ.

[61] Kirkpatrick DL, Craig RL, Bittel LR (1967). Evaluation of training. Training and Development Handbook.

[62] Lund F, Schultz JH, Maatouk I, Krautter M, Moltner A, Werner A, Weyrich P, Jünger J, Nikendei C (2012). Effectiveness of IV cannulation skills laboratory training and its transfer into clinical practice: a randomized, controlled trial. PloS One.

[63] Lynagh M, Burton R, Sanson-Fisher R (2007). A systematic review of medical skills laboratory training: where to from here?. Med Educ.

[64] Cook DA, Hatala R, Brydges R, Zendejas B, Szostek JH, Wang AT, Erwin PJ, Hamstra SJ (2011). Technology-enhanced simulation for health professions education: a systematic review and meta-analysis. Jama.

[65] Cook DA, Brydges R, Hamstra SJ, Zendejas B, Szostek JH, Wang AT, Erwin PJ, Hatala R (2012). Comparative effectiveness of technology-enhanced simulation versus other instructional methods: a systematic review and meta-analysis. Simul Healthc.

[66] McGaghie WC, Issenberg SB, Cohen ER, Barsuk JH, Wayne DB (2011). Does simulation-based medical education with deliberate practice yield better results than traditional clinical education? A meta-analytic comparative review of the evidence. Acad Med.

[67] Herrmann-Werner A, Nikendei C, Keifenheim K, Bosse HM, Lund F, Wagner R, Celebi N, Zipfel S, Weyrich P (2013). "Best practice" skills lab training vs. a "see one, do one" approach in undergraduate medical education: an RCT on students' long-term ability to perform procedural clinical skills. PloS One.

[68] Weyrich P, Celebi N, Schrauth M, Moltner A, Lammerding-Koppel M, Nikendei C (2009). Peer-assisted versus faculty staff-led skills laboratory training: a randomised controlled trial. Med Educ.

[69] Hudson JN, Tonkin AL (2008). Clinical skills education: outcomes of relationships between junior medical students, senior peers and simulated patients. Med Educ.

[70] Tolsgaard MG, Gustafsson A, Rasmussen MB, Hoiby P, Muller CG, Ringsted C (2007). Student teachers can be as good as associate professors in teaching clinical skills. Med Teach.

[71] Yu TC, Wilson NC, Singh PP, Lemanu DP, Hawken SJ, Hill AG (2011). Medical students-as-teachers: a systematic review of peer-assisted teaching during medical school. Adv Med Educ Pract.

[72] Secomb J (2008). A systematic review of peer teaching and learning in clinical education. J Clin Nurs.

[73] Ten Cate O, Durning S (2007). Dimensions and psychology of peer teaching in medical education. Med Teach.

[74] Lockspeiser TM, O'Sullivan P, Teherani A, Muller J (2008). Understanding the experience of being taught by peers: the value of social and cognitive congruence. Adv Health Sci Educ.

[75] Schmidt HG, Moust JH (1995). What makes a tutor effective? A structural-equations modeling approach to learning in problem-based curricula. Acad Med.

[76] Ker J, Hogg G, N M, Walsh K (2010). Cost effective simulation. Cost Effectiveness in Medical Education.

[77] Zendejas B, Wang AT, Brydges R, Hamstra SJ, Cook DA (2013). Cost: the missing outcome in simulation-based medical education research: a systematic review. Surgery.

[78] Brugger P, Threin M, Wolters M (2013). Hochschulen auf einen Blick.

[79] Meyer O, Pierre M, Breuer G (2013). Simulators don't teach – Lernprozesse und Simulation. Simulation in der Medizin.

[80] Kohn LT, Corrigan JM, Donaldson MS, Institute of Medicine (US) Committee on Quality of Health Care in America (2000). To Err is Human: Building a Safer Health System.

[81] Haluck RS, Krummel TM (1999). Simulation and virtual reality for surgical education. Surg Techn Int.

[82] Reznek M, Harter P, Krummel T (2002). Virtual reality and simulation: training the future emergency physician. Acad Emerg Med.

[83] Fischer MR, Bauer D, Mohn K (2015). Finally finished! National Competence Based Catalogues of Learning Objectives for Undergraduate Medical Education (NKLM) and Dental Education (NKLZ) ready for trial. GMS Z Med Ausbild.

